# Solid Fuel Exposure and Chronic Obstructive Pulmonary Disease in Never-Smokers

**DOI:** 10.3389/fmed.2021.757333

**Published:** 2021-12-22

**Authors:** HuanYu Long, ZhenZhen Xing, Di Chai, WeiMing Liu, YaQi Tong, YuXia Wang, YaLi Ma, MingMing Pan, Jia Cui, YanFei Guo

**Affiliations:** ^1^Department of Respiratory and Critical Care Medicine, Beijing Hospital, National Center of Gerontology, Institute of Geriatric Medicine, Chinese Academy of Medical Sciences, Beijing, China; ^2^The Key Laboratory of Geriatrics, Beijing Institute of Geriatrics, Beijing Hospital, National Center of Gerontology, National Health Commission, Institute of Geriatric Medicine, Chinese Academy of Medical Sciences, Beijing, China; ^3^Department of Intensive Care Medicine, Beijing Boai Hospital, Beijing, China; ^4^Rehabilitation Research Center, Beijing, China

**Keywords:** chronic obstructive pulmonary disease, never-smokers, prevalence, solid fuel, small airway dysfunction

## Abstract

**Background:** Chronic obstructive pulmonary disease (COPD) is a public health challenge globally. The burden of COPD is high in never-smokers but little is known about its causes. We aimed to find the prevalence and correlates of COPD in never-smokers, with a special focus on solid fuel exposure.

**Methods:** We conducted a cross-sectional study in Western China. COPD was defined by FEV1/FVC < lower limits of normal (LLN). Descriptive statistics and multivariable logistic regression were used for analyses.

**Results:** Six thousand two hundred and seventy one patients were enrolled between June 2015 and August 2016. The prevalence of COPD in never-smokers was 15.0% (95% confidence interval 14.1–15.9). The common independent predictors of COPD in never-smokers included age ≥60 years, exposure to solid fuel, living in a rural area and a history of tuberculosis. Participants with solid fuel exposure were 69% more likely to have COPD (adjusted odds ratio 1.69, 95% CI 1.41–2.04) than those without such exposure. In addition, we found a positive association between small airway dysfunction and solid fuel exposure (OR 1.35, 95% CI 1.18–1.53).

**Conclusions:** This study confirmed the substantial burden of COPD among never-smokers and also defined the risk factors for COPD in never-smokers. Furthermore, we found a positive association between solid fuel exposure and COPD or small airway dysfunction.

## Introduction

Chronic obstructive pulmonary disease (COPD) is a public health challenge globally, with high prevalence, morbidity and mortality (1, 2). Cigarette smoking is regarded as a major risk factor for COPD (3–5), but never-smokers comprise a substantial proportion of patients suffering from COPD (6). Indeed, it has been estimated that 25–45% of COPD patients are never-smokers (6, 7). The burden of COPD in never-smokers is thus substantial in developed and developing countries (7, 8).

The World Health Organization (WHO) estimates that about half of the global population depends on solid fuel for domestic cooking or heating (9). Burning of solid fuel produces large amounts of health-damaging chemicals (10, 11). Exposure to solid fuel has been linked to a range of adverse health outcomes including lung cancer, COPD and pneumonia in adults, especially among women (12–14).

Numerous studies have been carried out to evaluate the prevalence and risk factors of COPD, while fewer studies have focused solely on never-smokers. In a recent nationwide cross-sectional study in China, more than half of the COPD patients were never-smokers (15). In Tibet Autonomous Region (Tibet) and Xinjiang Uygur Autonomous Region (Xinjiang), solid fuel were generally used, with more than half of households relying on solid fuel for cooking or heating (16). We aimed to assess the prevalence and correlates of COPD among never-smokers in Tibet and Xinjiang, with a particular focus on solid fuel exposure.

## Methods

### Study Setting

We performed a cross-sectional study on the prevalence and determinants of COPD among people living in Xinjiang and Tibet, China. Xinjiang and Tibet are the largest and second largest provincial-level autonomous regions in China, located along the western border of China. The Institutional Review Board and ethics committee of Beijing Hospital (2013BJYYEC-042C-01) approved the protocol of this study.

### Participants

Detailed methods of the study have been described previously (17). Between June 2015 and August 2016, a multistage stratified sampling procedure was conducted to select a representative sample of subjects living in Xinjiang and Tibet. First, 13 districts or counties were selected in both urban (2 districts) and rural areas (11 counties) using the probability proportional to size method. Second, a simple random sampling method was adopted; two streets or townships were selected from each district or county, and three communities or village communities were selected from each street or township. Third, we randomly selected individuals aged 15 years or older from the selected communities or villages. The proportion of samples from each gender and age group was based on the 2010 census of Chinese population. We chose only one participant from each household.

We included permanent residents (who had lived at their current residence for 1 year or longer) aged ≥15 years for this analysis. Exclusion criteria were as follows: pregnancy; myocardial infarction or cerebrovascular accident experienced during the previous 3 months; heart rate >120 beats/min or blood pressure >180/120 mmHg; and being physically incapable of undergoing a spirometry test, such as due to recently undergoing thoracic, abdominal, or eye surgery, or retinal detachment. Each participant received detailed information about the study and study methods, and provided written informed consent before data collection.

### Operational Definition

Trained and certified technicians performed pulmonary function tests for all participants. We performed daily calibration with a 3 L syringe and also recorded ambient temperature, humidity and altitude daily. Forced expiratory volumes were measured using the MasterScreen^TM^ Pneumo PC spirometer (CareFusion, Yorba Linda, CA) before and 20 min after the administration of 400 μg of salbutamol through a 500 ml spacer. Each spirogram was centrally reviewed and scored based on the American Thoracic Society and European Respiratory Society (ATS/ERS) acceptability and reproducibility criteria (18). We defined: (1) COPD as a post-bronchodilator FEV1/FVC below the lower limit of normal (LLN) of the Global Lung Function Initiative (GLI) 2012 multiethnic equations (19); and (2) small airway dysfunction on the basis of at least two of the following three indicators of lung function being <65% of the predicted level: maximal mid-expiratory flow (MMEF), forced expiratory flow (FEF) 50% and FEF 75% (20). We calculated the ratios of observed to predicted FEV1 based on GLI 2012 multiethnic equations and used them to stage the degree of obstruction (GOLD stage I, ≥80% predicted; GOLD stage II, ≥50– <80% predicted; GOLD stage III, ≥30– <50% predicted; and GOLD stage IV, <30% predicted) (19). Moreover, we used a post-bronchodilator FEV1/FVC < 0.70, according to 2021 Global Initiative for Chronic Obstructive Lung Disease (GOLD) guidelines (1), define COPD in a sensitivity analysis.

Never-smokers were defined as a participant who has never smoked, or who has smoked <100 cigarettes in his or her lifetime (21). Passive smoking was defined as inhalation of smoke by never-smokers who lived with smokers. Solid fuel was defined based on whether the participant had used an open fire with charcoal, coal, coke, wood, crop residues or dung as the primary means of cooking or heating for more than 6 months in their lifetime. History of tuberculosis was defined as a positive answer to the question “Has a doctor or other health care provider ever told you that you had tuberculosis?” Peripheral oxygen saturation was measured by pulse-oximetry (PHILIPS DB12) before performing spirometry.

Participants were considered to have: (1) chronic cough if they have a cough for most of the day for as much as 3 consecutive months during a year; (2) chronic phlegm if they bring up phlegm for most of the day for as much as 3 consecutive months during a year; (3) recurrent wheezing if their chest (lungs) ever sounded wheezy (whistling sound); (4) dyspnea in daily life if they were troubled by shortness of breath when hurrying on level ground, walking up a slight incline, walking at their own pace on level ground or being breathless when dressing/undressing or going out; and (5) at least one symptom if they had at least one of either chronic cough, chronic phlegm, recurrent wheezing or dyspnea in daily life.

### Statistical Analysis

Descriptive statistics such as count and percentage for categorical variables and mean and standard deviation for continuous variables were determined separately for men and women. We conducted independent Student's *t-*test and χ^2^ test for continuous and categorical variables, respectively. We performed univariable logistic regression to determine the distribution of the study subjects by the independent variable of interest and to see crude association. Meanwhile, we used multivariable logistic regression analysis adjusted for sex, age, body mass index (BMI), education, education, residence, history of tuberculosis, exposure to solid fuel, exposure to passive smoking, and exposure to dust or chemicals in the workplace with adjusted odds ratio. In the sensitivity analyses, we applied a fixed cut-off to determine COPD.

All statistical analyses were performed using R with ggplot2, forestplot and ordinal packages (www.r-project.org). For all tests, the *p*-value was set to <0.05 to determine significance.

## Results

### Population Characteristics

Between June 2015 and August 2016, a total of 12,991 individuals were randomly selected and invited to participate in the survey, of whom 6,271 never-smokers consented and completed the study (Supplementary Figure 1).

The characteristics of the 6,271 participants are presented in Table 1. The average age (SD) among the participants was 39.7 (15.6) years, and the mean BMI was 24.2 (4.1) Kg/m^2^, with 68.1% of the subjects being women. Overall, 83.8% of participants were lived in rural areas, 70.8% were exposed to solid fuel, 21.1% were exposed to passive smoking, 3.9% had a history of tuberculosis.

**Table 1:** Sociodemographic Characteristics in participants.

	**Men**	**Women**	**Overall**
**Subjects** ***n***	2,000	4,271	6,271
**Mean age, years, mean** ± **SD**	37.8 ± 16.8	40.6 ± 14.9	39.7 ± 15.6
**BMI, kg/m**^**2**^**, mean** ± **SD**	24.0 ± 3.9	24.2 ± 4.2	24.2 ± 4.1
**Living in a rural area**, ***n*** **(%)**	1,637 (81.8)	3,615 (84.6)	5,252 (83.8)
**Education**, ***n*** **(%)**			
Primary school or less	748 (37.4)	2,138 (50.1)	2,886 (46.0)
Middle school or high school	866 (43.3)	1,543 (36.1)	2,409 (38.4)
College and higher	386 (19.3)	590 (13.8)	976 (15.6)
**Risk factors for COPD**, ***n*** **(%)**			
Solid fuel exposure	1,275 (63.8)	3,167 (74.2)	4,442 (70.8)
Passive smoking^*^	269 (14.1)	1,010 (24.3)	1,279 (21.1)
History of tuberculosis	80 (4.0)	162 (3.8)	242 (3.9)
Exposure to dust or chemicals in the workplace^†^	135 (7.0)	446 (11.0)	581 (9.7)
**Spirometry, %, mean** **±** **SD**			
Pre-BD FEV1 %pred	93.5 ± 20.1	95.6 ± 22.7	94.9 ± 21.9
Post-BD FEV1 %pred	95.5 ± 20.9	97.8 ± 22.8	97.1 ± 22.3
Pre-BD FVC %pred	77.2 ± 17.1	80.1 ± 18.6	79.2 ± 18.2
Post-BD FVC %pred	78.9 ± 17.7	81.9 ± 18.7	80.9 ± 18.4
Pre-BD FEV1/FVC %	80.9 (12.0)	81.1 (10.8)	81.0 (11.2)
Post-BD FEV1/FVC %	82.6 (11.7)	82.9 (10.2)	82.8 (10.7)
**Oxyhemoglobin saturation, mean** ± **SD**^‡^	92.8 ± 5.3	93.5 ± 5.5	93.3 ± 5.4

*Data are presented as n, n (%) or mean ± SD. BMI, body mass index; BD, bronchodilator; COPD, chronic obstructive pulmonary disease; FVC: forced vital capacity; FEV1: forced expiratory volume in 1 s; pred, predicted;*

* *92 missing values in men and 116 missing values in women;*

† *84 missing values in men and 209 missing values in women;*

‡ *57 missing values in men and 100 missing values in women*.

### Epidemiology of COPD

The overall prevalence of LLN-defined COPD was 15.0% (95% CI 14.1–15.9) in the never-smoking population aged 15 years or older (Table 2). There was no difference in COPD prevalence (*P* = 0.94) between men and women. The age-specific prevalence of COPD rose significantly with increasing age (*P* < 0.05 for both men and women; Supplementary Figure 2). It reached 17.0% (95% CI 15.6–18.3) among those aged ≥40 years. COPD prevalence was higher in rural residents (15.9%, 95% CI 14.9–16.9) than in urban residents (10.5%, 8.7–12.5; *P* < 0.001; Table 2). The prevalence of COPD was 15.7% (95% CI 14.4–17.1) in primary school or less, 15.7% (95% CI 14.3–17.2) in middle school or high school, and 11.2% (95% CI 9.3–13.3) in college and higher. Participants with history of tuberculosis exposure had a higher prevalence of COPD than those without it (20.7%, 95% CI 15.7–26.3) vs. 14.8%, 95% CI 13.9–15.7); *P* < 0.02; Table 2).

**Table 2:** Prevalence of COPD in participants.

	**Overall**		**Men**		**Women**	
	**Cases/total (n/N)**	**Prevalence of COPD (95% CI)**	**Cases/total (n/N)**	**Prevalence of COPD (95% CI)**	**Cases/total (n/N)**	**Prevalence of COPD (95% CI)**
**Prevalence**	942/6,271	15.0% (14.1–15.9)	299/2,000	15.0% (13.4–16.6)	643/4,271	15.1% (14.0–16.2)
**Age, years**						
15–39	424/3,216	13.2% (12.0–14.4)	133/1,152	11.5% (9.8–13.5)	291/2,064	14.1% (12.6–15.7)
40–49	219/1,417	15.5% (13.6–17.4)	63/378	16.7% (13.1–20.8)	156/1,039	15.0% (12.9–17.3)
50–59	138/920	15.0% (12.8–17.5)	31/236	13.1% (8.1–18.1)	107/684	15.6% (13.0–18.6)
60+	161/718	22.4% (19.4–25.7)	72/234	30.8% (24.9–37.1)	89/484	18.4% (15.0–22.1)
*P*-value for trend		<0.0001		<0.0001		0.02151
**BMI, kg/m** ^**2**^						
<18.5 (underweight)	51/396	12.9% (9.7–16.6)	17/138	12.3% (7.3–19.0)	34/258	13.2% (9.3–17.9)
18.5–24.9 (normal weight)	509/3,405	14.9% (13.8–16.2)	152/1075	14.1% (12.1–16.4)	357/2330	15.3% (13.9–16.8)
≥25.0 (overweight and obesity)	382/2,470	15.5% (14.1–17.0)	130/787	16.5% (14.0–19.3)	252/1683	15.0% (13.3–16.8)
*P*-value for trend		0.2436		0.0937		0.8001
**Education**						
Primary school or less	454/2,886	15.7% (14.4–17.1)	134/748	17.9% (15.2–20.9)	320/2138	15.0% (13.5–16.6)
Middle school or high school	379/2,409	15.7% (14.3–17.2)	129/866	14.9% (12.6–17.4)	250/1543	16.2% (14.4–18.1)
College and higher	109/976	11.2% (9.3–13.3)	36/386	9.3% (6.6–12.7)	73/590	12.4% (9.8–15.3)
*P*-value for trend		0.0045		0.0002		0.4021
**Residence**						
Urban	107/1,019	10.5% (8.7–12.5)	26/363	7.2% (4.7–10.3)	81/656	12.3% (9.9–15.1)
Rural	835/5,252	15.9% (14.9–16.9)	273/1637	16.7% (14.9–18.6)	562/3615	15.5% (14.4–16.8)
*P*-value		<0.0001		<0.0001		0.0405
**Solid fuel exposure**						
Yes	759/4,442	17.1% (16.0–18.2)	227/1275	17.8% (15.7–20.0)	532/3167	16.8% (15.5–18.1)
No	183/1,829	10.0% (8.7–11.5)	72/725	9.9% (7.9–12.3)	111/1104	10.1% (8.3%−12.0)
*P*-value		<0.0001		<0.0001		<0.0001
Passive smoking^*^						
Yes	192/1,279	15.0% (13.1–17.1)	38/269	14.1% (10.2–18.9)	154/101	15.2% (13.1–17.6)
No	718/4,784	15.0% (14.0–16.1)	248/1639	15.1% (13.4–17.0)	470/3145	14.9% (13.7–16.2)
*P-*value		1		0.7371		0.854
**History of tuberculosis**						
Yes	50/242	20.7% (15.7–26.3)	15/80	18.8% (10.9–29.0)	35/162	21.6% (15.5–28.7)
No	892/6,029	14.8% (13.9–15.7)	284/1920	14.8% (13.2–16.5)	608/4109	14.8% (13.7–15.9)
*P*-value		0.0158		0.4163		0.0235
**Exposure to dust or chemicals in the workplace** ^†^						
Yes	73/581	12.6% (10.0–15.5)	16/135	11.9% (6.9–18.5)	57/446	12.8% (9.8–16.2)
No	821/5,397	15.2% (14.3–16.2)	268/1781	15.0% (13.4–16.8)	553/3613	15.3% (14.1–16.5)
*P*-value		0.1012		0.3778		0.1809

*BMI, body mass index; COPD, chronic obstructive pulmonary disease; CI, confidence interval;*

* *208 missing values;*

† *293 missing values*.

We also performed a sensitivity analysis using an age-specific fixed cut-off to define COPD. The overall prevalence of spirometry-defined COPD was 10.6% (95% CI 9.9–11.4). Men had a higher prevalence (11.8%, 95% CI 10.4–13.3) than women (10.1%, 95% CI 9.2–11.0; *P* < 0.05). Supplementary Table 1 presents the severity of COPD by GOLD grades. Approximately 34.5% COPD patients self-reported typical symptoms, such as frequent cough, sputum, recurrent wheezing, or dyspnea in daily life (Supplementary Table 2).

### Predictors of COPD

**Figure 1:**
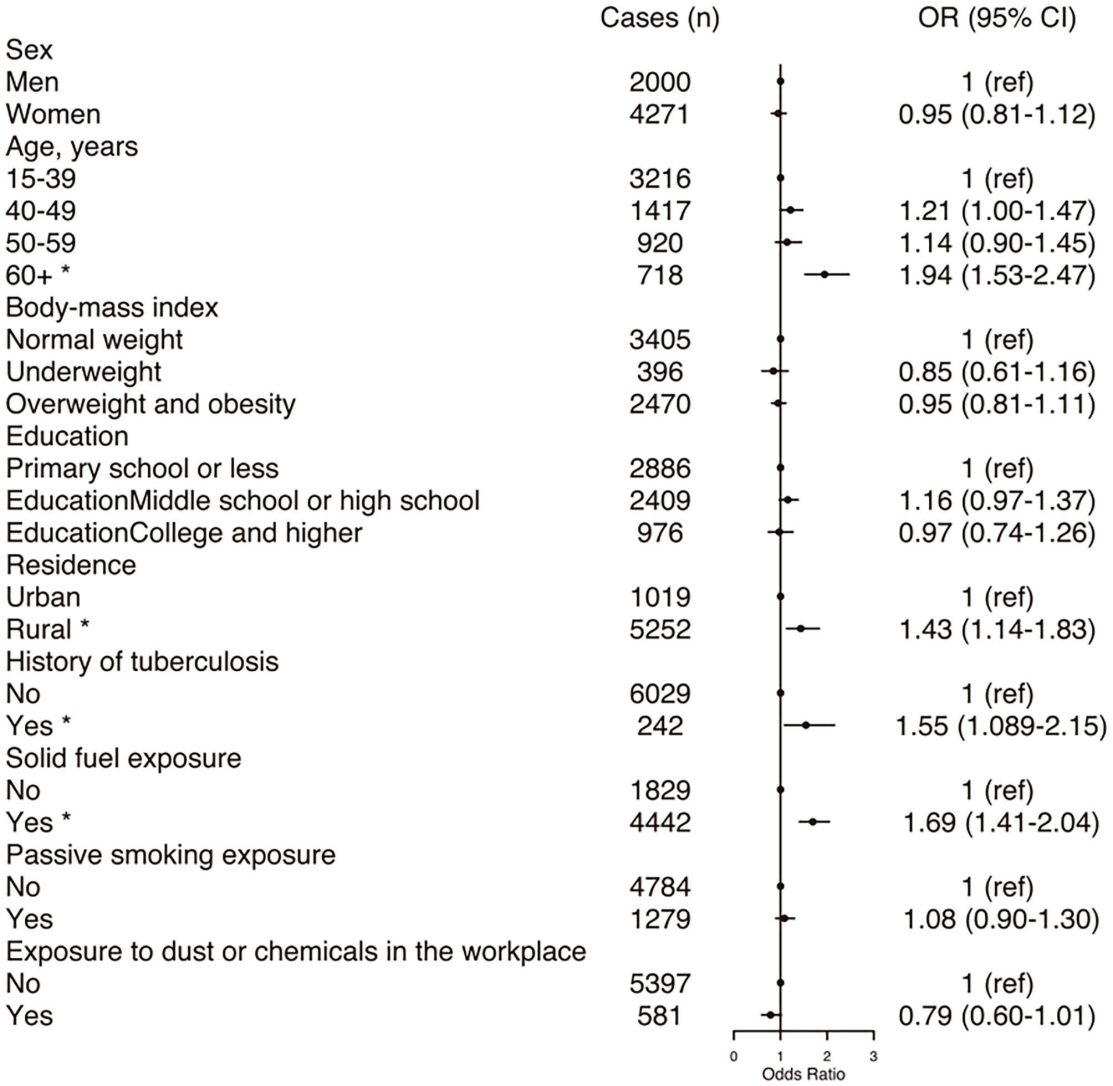
Forest plot showing OR for COPD in participants. COPD, chronic obstructive pulmonary disease; CI, confidence interval; OR, odd ratio. Each circle represents an OR. The horizontal lines indicate 95%CI. Adjusted ORs for COPD by sex, age, body mass index, education, residence, history of tuberculosis, exposure to solid fuel, exposure to passive smoking, exposure to dust or chemicals in the workplace. *P < 0.05.

Figure 1 displays the adjusted OR and 95% CI from the multivariable logistic regression analysis to determine the COPD-associated risk factors. For never-smokers with COPD, the common independent association was age ≥60 years, exposure to solid fuel, living in a rural area and a history of tuberculosis.

### Characteristics of Participants by Solid Fuel Exposure

**Figure 2:**
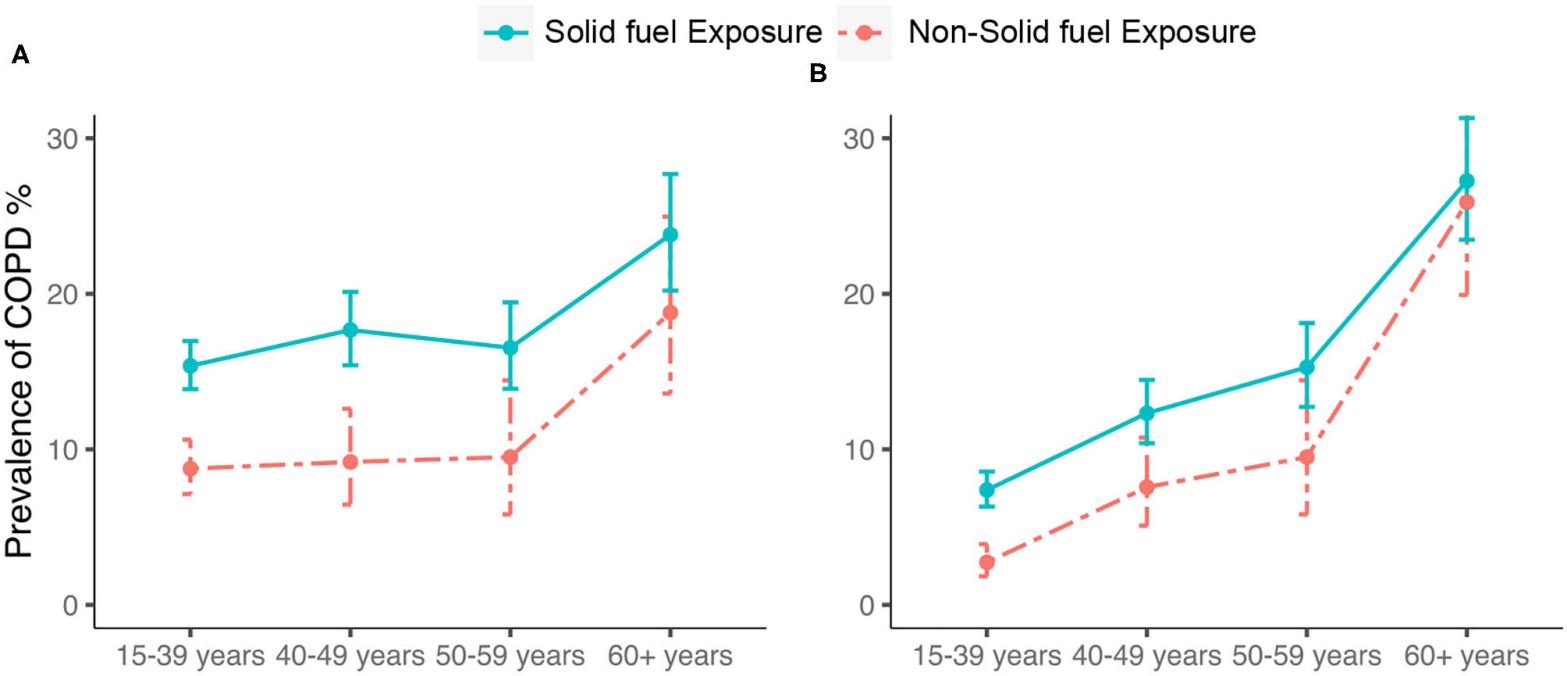
Prevalence and corresponding 95%CI of COPD by **(A)** LLN-defined COPD and **(B)** spirometry- defined COPD. COPD, chronic obstructive pulmonary disease; CI, confidence interval; Bars represent the prevalence and error bars the 95%CI.

Participants with solid fuel exposure had a higher prevalence of COPD than those without it (17.1% (95% CI 16.0–18.2) vs. 10.0% (95% CI 8.7–11.5); *P* < 0.0001) (Table 2). The age-stratified prevalence of COPD was significantly higher in participants with solid fuel exposure than in those without it, and the fixed cut-off used did not change this result (Figure 2).

**Figure 3:**
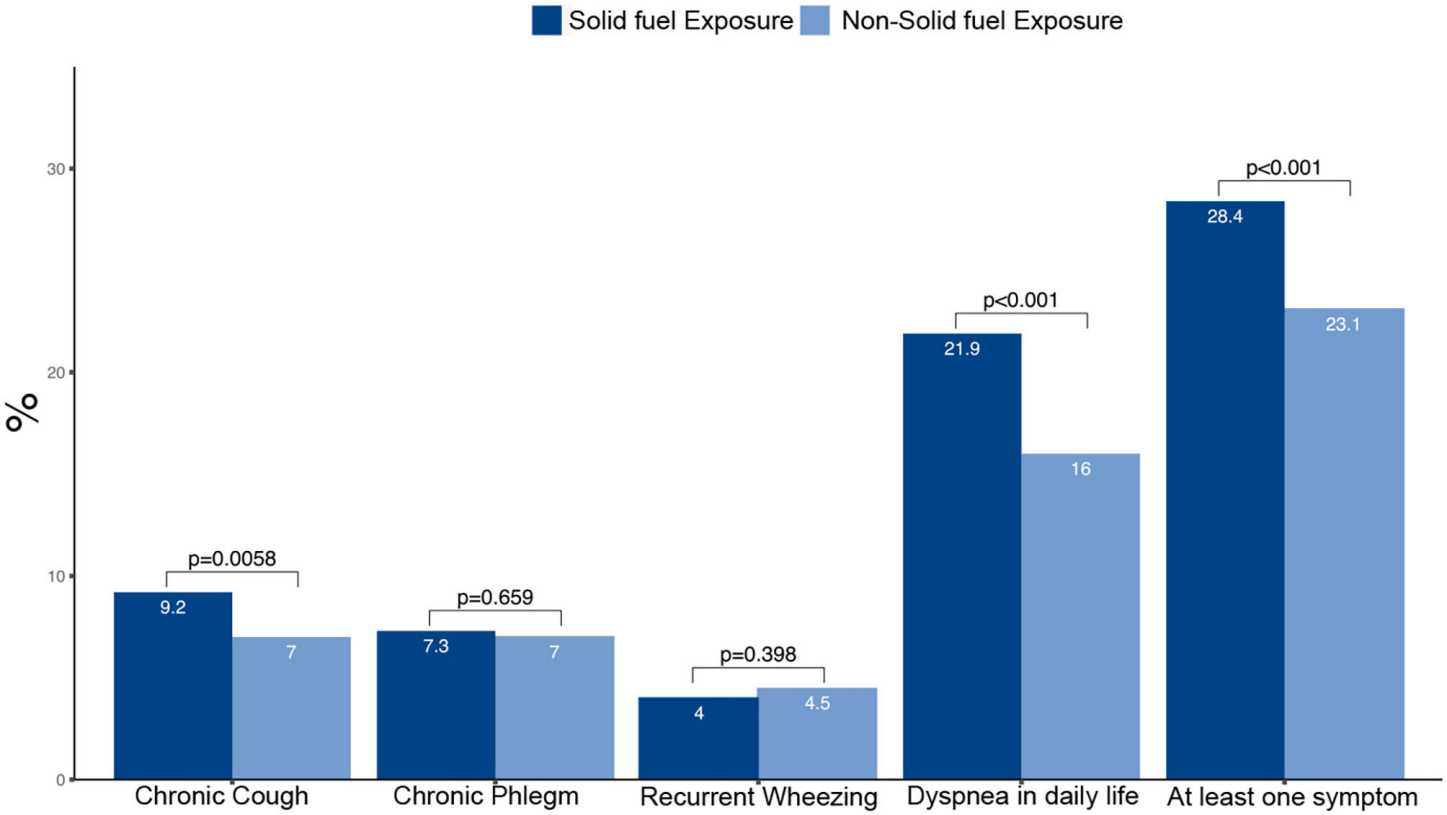
Prevalence and corresponding 95%CI of symptoms by solid fuel exposure.

Participants with solid fuel exposure tended to be older, women, less educated and living in rural areas compared with those without such exposure (Table 3). On average, participants with solid fuel exposure had lower pre- and post-bronchodilator FEV1, FVC and FEV1/FVC than those without such exposure. Participants with solid fuel exposure had higher frequencies of chronic cough, dyspnea in daily life and at least one symptom when compared with those without such exposure (Figure 3).

**Table 3:** Characteristics of participants by solid fuel exposure.

	**Non-solid fuel exposure**	**Solid fuel exposure**	***P*-value**
**Subjects** ***n***	1,829	4,442	
**Women**, ***n*** **(%)**	1,104 (60.4)	3,167 (71.3)	<0.001
**Living in a rural area**, ***n*** **(%)**	1,270 (69.4)	3,982 (89.6)	<0.001
**Age, years**, ***n*** **(%)**			
15–39	1,062 (58.1)	2,154 (48.5)	<0.001
40–49	370 (20.2)	1,047 (23.6)	
50–59	200 (10.9)	720 (16.2)	
60+	197 (10.8)	521 (11.7)	
**BMI, kg/m**^**2**^, ***n*** **(%)**			
<18.5 (underweight)	1,024 (56.0)	2,381 (53.6)	0.179
18.5–24.9 (normal weight)	117 (6.4)	279 (6.3)	
≥25.0 (overweight and obesity)	688 (37.6)	1,782 (40.1)	
**Education**, ***n*** **(%)**			
Primary school or less	616 (33.7)	2,270 (51.1)	<0.001
Middle school or high school	638 (34.9)	1,771 (39.9)	
College and higher	575 (31.4)	401 (9.0)	
**Spirometry, %, mean** ± **SD**			
Pre-BD FEV1/FVC %	83.0 (10.4)	80.2 (11.3)	<0.001
Post-BD FEV1/FVC %	84.8 (9.6)	82.0 (11.0)	<0.001
Pre-BD FEV1 %pred	96.0 ± 21.6	94.5 ± 22.1	0.0104
Post-BD FEV1 %pred	97.9 ± 21.6	96.7 ± 22.5	0.0519
Pre-BD FVC %pred	80.2 ± 18.1	78.7 ± 18.2	0.00228
Post-BD FVC %pred	81.8 ± 18.0	80.6 ± 18.6	0.0147
Pre-BD MMEF %pred^*^	74.8 ± 30.5	70.4 ± 31.7	<0.001
Pre-BD FEF50 %pred^†^	84.6 ± 28.7	79.5 ± 29.9	<0.001
Pre-BD FEF75 %pred^‡^	89.4 ± 46.5	80.3 ± 46.5	<0.001

*Data are presented as n, n (%) or mean ± SD. BMI, body mass index; BD, bronchodilator; FVC: forced vital capacity; FEV1: forced expiratory volume in 1 s; FEF, forced expiratory flow; MMEF, maximal mid-expiratory flow; pred, predicted;*

* *64 missing values in non-solid fuel exposure and 169 missing values in solid fuel exposure;*

† *11 missing values in non- solid fuel exposure and 47 missing values in solid fuel exposure;*

‡ *11 missing values in non- solid fuel exposure and 48 missing values in solid fuel exposure*.

Participants with solid fuel exposure were more likely to have COPD than those without such exposure. The adjusted OR and 95% CI for the association between solid fuel exposure and COPD was stronger in women (adjusted OR 1.74, 95% CI 1.39–2.21) than in men (1.64, 1.21–2.24) (Table 4).

**Table 4:** Association of COPD, small airway dysfunction, and symptoms with solid fuel exposure.

	**Overall**	**Men**	**Women**
	**Adjusted OR (95% CI)**	**Adjusted OR (95% CI)**	**Adjusted OR (95% CI)**
COPD (FEV1/FVC < LLN)	1.69 (1.41–2.04)^*^	1.64 (1.21–2.24)^*^	1.74 (1.39–2.21)^*^
COPD (FEV1/FVC <0.7)	1.74 (1.40–2.18)^*^	1.62 (1.14–2.34)^*^	1.87 (1.40–2.52)^*^
Small airway dysfunction	1.35 (1.18–1.53)^*^	1.28 (1.02–1.61)^*^	1.41 (1.20–1.65)^*^
Chronic cough	1.35 (1.18–1.53)^*^	1.63 (1.08–2.51)^*^	1.52 (1.14–2.06)^*^
Chronic phlegm	1.25 (0.98–1.61)	1.17 (0.78–1.77)	1.33 (0.97–1.85)
Recurrent wheezing	0.912 (0.67–1.25)	1.45 (0.75–2.87)	0.81 (0.57–1.16)
Dyspnea in daily life	1.72 (1.45–2.05)^*^	1.50 (1.11–2.05)^*^	1.84 (1.49–2.27)^*^
At least one symptom	1.72 (1.47–2.01)^*^	1.61 (1.23–2.11)^*^	1.79 (1.48–2.17)^*^

*COPD, chronic obstructive pulmonary disease; CI, confidence interval; OR, odd ratio; LLN, lower limits of normal; Adjusted ORs by sex, age, body mass index, education, residence, history of tuberculosis, exposure to passive smoking, exposure to dust or chemicals in the workplace;*

* *P < 0.05*.

Participants with solid fuel exposure were more likely to have chronic cough, dyspnea in daily life and at least one symptom than those without such exposure (Table 4). Participants with solid fuel exposure were 72% more likely to have at least one symptom (cough, sputum, wheeze, dyspnea) than participants without such exposure (OR 1.72, 95% CI 1.47–2.01).

### Solid Fuel and Small Airway Dysfunction

**Figure 4:**
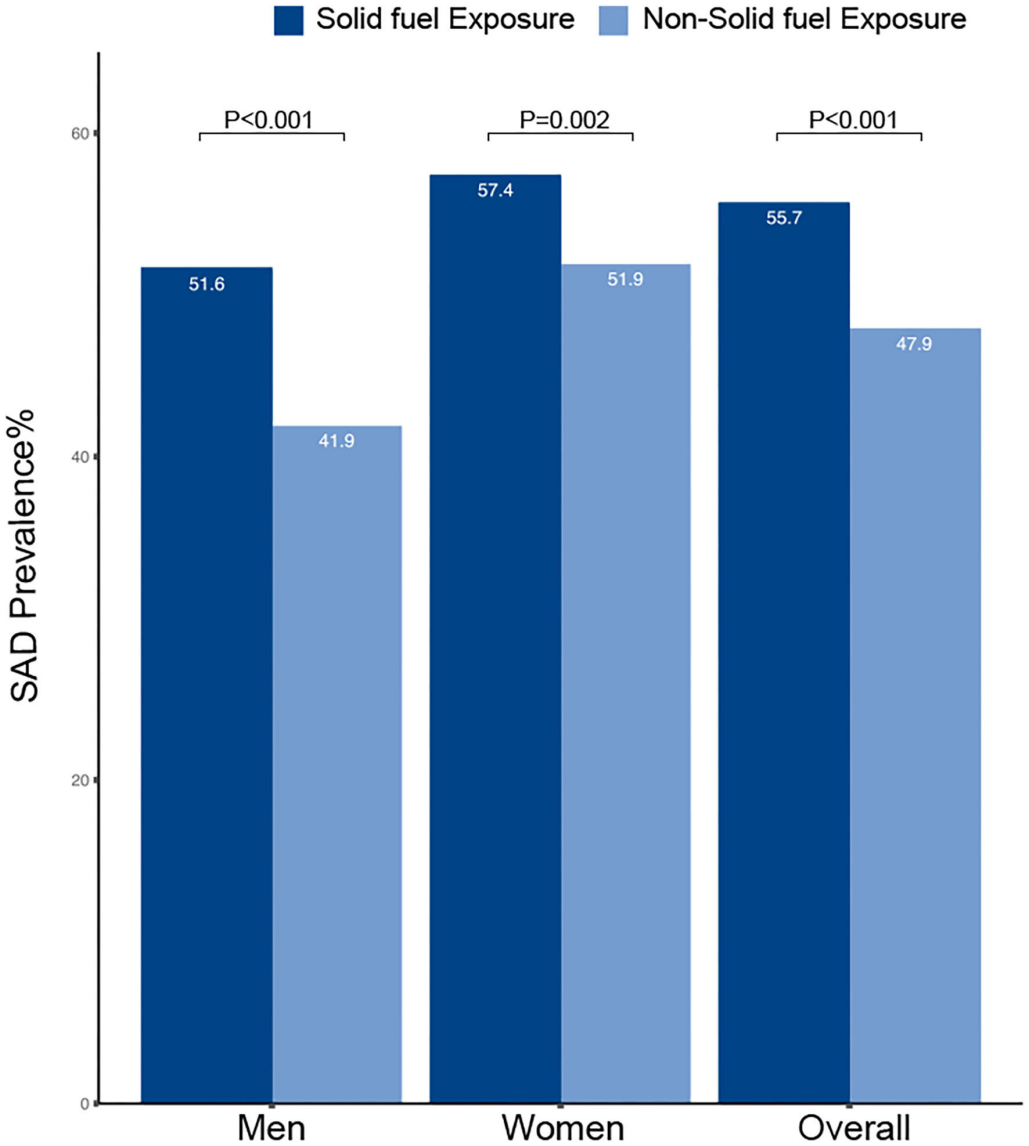
Prevalence and corresponding 95%CI of SAD by solid fuel exposure. CI, confidence interval; SAD, small airway dysfunction.

The prevalence of small airway dysfunction was higher in participants with solid fuel exposure than in those without such exposure (55.7% vs. 47.9%; *P* < 0.001) (Figure 4). The sex-stratified prevalence of COPD was significantly higher in participants with solid fuel exposure than in those without it. Participants with solid fuel exposure were also more likely to have small airway dysfunction than those without such exposure (OR 1.35, 95% CI 1.18–1.53) (Table 4).

## Discussion

In this study, we analyzed the prevalence of COPD and its risk factors among never-smokers in Xinjiang and Tibet, with a special focus on solid fuel exposure. Both COPD prevalence and solid fuel exposure were significantly high among never-smokers. The factors independently associated with COPD were age ≥60 years, exposure to solid fuel, living in a rural area and a history of tuberculosis. In addition, we found a positive association between solid fuel exposure and respiratory symptoms or small airway dysfunction. Our results highlight the substantial burden of COPD among never-smokers and confirm the increasingly acknowledged impact of solid fuel on respiratory health.

Based on BOLD and other large scale epidemiological studies, it is estimated that the global prevalence of COPD is about 11.7%, which increased 68.9% between 1990 and 2010 (22). Although smoking is the leading cause of COPD in the world, an estimated 25–45% of patients with COPD have never smoked (7). Never-smokers with COPD are an important population that is increasing but remains understudied. The China Kadoorie Biobank (CKB) study (8) showed that the prevalence of COPD was 5.9% for women and 5.2% for men, but the prevalence varies widely by region. The prevalence of COPD in our study was 15.0% in western China, which is consistent with the CKB study of COPD prevalence in Sichuan province, which is a high COPD prevalence that they cannot explain” (8). Sichuan province borders Tibet to the west, and they both belong to western China. Our study provides further insight into the prevalence of COPD in never-smokers populations in western China. The prevalence of COPD in never-smokers was in line with the BOLD study (18.2%) (23). Although this rate was higher than in studies from developed countries, such as the OLIN study (6.9%) and the CanCOLD study (6.4%), the rates of solid fuel use and tuberculosis are considerably lower than previously reported in developing countries (24, 25).

About 3 billion people worldwide use solid fuel for cooking or heating (7). Despite the decrease in the percentage of people using solid fuels, the absolute population exposed to solid fuel is more than three times greater than those smoking daily (12). Solid fuel causes an estimated 3.5–4 million deaths every year and more than 90% of solid fuel-related deaths occur in developing countries, mainly in Asia and Africa (11). International studies showed that participants with solid fuel exposure were 41% more likely to have COPD than those without such exposure (26). In addition, a cohort study revealed that long-term exposure to solid fuel for cooking was associated with about 10% excess risk of hospitalization or death due to COPD (27). The relationship between solid fuel exposure and COPD reported in our study was positive and the association was stronger than the results of previous analyses probably because our analysis was conducted in never-smokers (26) and the proportion of subjects exposed to solid fuel in our study was higher (70.8%) than that reported in these previous surveys. Solid fuel use has the potential to adversely affect the entire population, but the effects of solid fuel may be more apparent in never-smokers, in whom exposure to tobacco is not a competing risk factor (28).

The small airways are the major and earliest site of airflow limitation. Pathological studies suggest that loss of small airways prior to the development of abnormal spirometry or emphysema in COPD patients (29, 30). Recently, a cross-sectional study highlights that small airway disease is a pathological feature in mild and moderate COPD. Previous studies suggested that biomass smoke exposed patients demonstrated a distinct profile of small airways disease when compared to tobacco smoke exposed patients (31, 32). Our study showed that the prevalence of small airway dysfunction is higher in participants with solid fuel exposure than in those without it. We also found that participants with soldi fuel exposure had lower levels of pre- and post-bronchodilator FEV1, FVC and FEV1/FVC ratio, reflecting worse lung function among never-smokers. Previous studies found exposure-response relationships between solid fuel and lung function (33, 34). Individuals exposed to solid fuel had a higher prevalence of small airway dysfunction and lower lung function, suggesting that chronic inflammation from solid fuel exposure is associated with the development of chronic airway disease. Although the pathogenic mechanisms behind the harm caused by solid fuel are not yet fully understood, solid fuel is considered to enhance lung inflammation, alter the innate immune response and promote an oxidative stress state (35).

Our study emphasizes the importance of solid fuel reduction strategies, as public health intervention, which can reduce the burden of COPD. Previous studies showed that the adoption of clean fuel may be beneficial for preventing COPD (36). Meanwhile, the elevated risks associated with historical solid fuel use may still be attenuated by switching to clean fuels later in life (27, 36). A retrospective cohort study reported significantly lower risks of COPD in lifelong coal users who adopted a ventilated stove for cooking than in those who did not (37). This should encourage greater efforts to facilitate universal access to clean energy, as promoted by the United Nations Sustainable Development Goal (38).

Previous pulmonary tuberculosis was reported to be associated with airway fibrosis, and may lead to lung scarring and airway damage (7, 39). The BOLD and PLATINO studies revealed stronger associations between self-reported history of tuberculosis and COPD in people who had never smoked (6, 39). Similarly, in our study, the history of tuberculosis was closely related to the diagnosis of COPD among never-smokers. Therefore, effective measures for preventing tuberculosis are important in the prevention of COPD in this population.

Living in a rural area and increasing age were associated with a higher rate of COPD in our study, which is consistent with previous findings. For example, rural residence was previously reported to be an independent risk factor for COPD among never-smokers (28). The association between COPD and older age among never-smokers could be attributable to the accumulative effects of risk factors over time and the age-related weakening of respiratory muscles that leads to decreased FEV1/FVC, as determined by standardized spirometry tests (40).

There are some limitations to our study. First, these data were cross-sectional and we are unable to draw firm conclusions in terms of causation and measure other potential risk factors. Second, some analyses were limited by the design of the questionnaire, which was intended to be comprehensive and easy to administer, but in some cases prevented optimal detailed data collection. We were unable to quantify direct exposure to solid fuel beyond self-reported questionnaires. Third, with a male-to-female ratio of 1 to 2 in our study sample, one may argue that the results could not be generalizable to men. However, in the gender-specific analysis (with 2,000 men), we found no evidence of heterogeneity. Fourth, the diagnosis of small airway dysfunction in this study was entirely based on spirometry, which could be less accurate than pathological examination and imaging techniques. Finally, those with asthma were not excluded from the study population, which might cause an over-estimate of COPD prevalence in younger age groups.

## Conclusions

In conclusion, this study confirmed the substantial burden of COPD among never-smokers. Our data suggested that, apart from exposure to solid fuel, history of tuberculosis, living in a rural area and older age are associated with an increased risk of COPD among never-smokers. Furthermore, we found a positive association between solid fuel exposure and COPD or small airway dysfunction.

## Data Availability Statement

The original contributions presented in the study are included in the article/Supplementary Material, further inquiries can be directed to the corresponding author.

## Ethics Statement

The studies involving human participants were reviewed and approved by the Institutional Review Board and Ethics Committee of Beijing Hospital (2013BJYYEC- 042C-01). Written informed consent to participate in this study was provided by the participants' legal guardian/next of kin.

## Author Contributions

HL, ZX, and YG conceived the study. HL, ZX, DC, WL, YT, YW, YM, JC, and MP collected data. HL, ZX, DC, and WL analyzed and interpreted data. HL and ZX drafted the manuscript. HL, ZX, and YG revised the manuscript. YG obtained funding and supervised the study. All authors contributed to the article and approved the submitted version.

## Funding

This study was supported by National Key Research and Development Program of China (2018YFC1315101), Beijing Hospital Clinical Research 121 Project (BJ-2018-199).

## Conflict of Interest

The authors declare that the research was conducted in the absence of any commercial or financial relationships that could be construed as a potential conflict of interest.

## Publisher's Note

All claims expressed in this article are solely those of the authors and do not necessarily represent those of their affiliated organizations, or those of the publisher, the editors and the reviewers. Any product that may be evaluated in this article, or claim that may be made by its manufacturer, is not guaranteed or endorsed by the publisher.

## Abbreviations

ATS/ERS: American Thoracic Society and European Respiratory Society
BMI: body mass index
COPD: chronic obstructive pulmonary disease
CI: confidence interval
FEF: forced expiratory flow
FEV1: forced expiratory volume in 1 s
FVC: forced vital capacity
GOLD: global initiative for chronic obstructive lung disease
GLI: global lung function initiative
LLN: lower limits of normal
MMEF: maximal mid-expiratory flow
OR: odds ratio
SD: standard deviation
WHO: world health organization.

## References

[1] Global Strategy for the Diagnosis Management Management prevention of COPD. Global Initiative for Chronic Obstructive Lung Disease (GOLD). (2021). Available online at: http://www.goldcopd.org/ (accessed November 2, 2021).

[2] GBD 2017 Causes of Death Collaborators. Global, regional, and national age-sex-specific mortality for 282 causes of death in 195 countries and territories, 1980-2017: a systematic analysis for the global burden of disease study 2017. Lancet. (2018) 392:1736–88. 30496103 10.1016/S0140-6736(18)32203-7 PMC6227606

[3] Burney P Jithoo A Kato B Janson C Mannino D Nizankowska-Mogilnicka E. Chronic obstructive pulmonary disease mortality and prevalence: the associations with smoking and poverty–a BOLD analysis. Thorax. (2014) 69:465–73. 10.1136/thoraxjnl-2013-204460 24353008 PMC3995258

[4] de Marco R Accordini S Marcon A Cerveri I Antó JM Gislason T. Risk factors for chronic obstructive pulmonary disease in a European cohort of young adults. Am J Respir Crit Care Med. (2011) 183:891–7. 10.1164/rccm.201007-1125OC 20935112

[5] Lindberg A Jonsson AC Rönmark E Lundgren R Larsson LG Lundbäck B. Prevalence of chronic obstructive pulmonary disease according to BTS, ERS, GOLD and ATS criteria in relation to doctor's diagnosis, symptoms, age, gender, smoking habits. Respiration. (2005) 72:471–9. 10.1159/000087670 16210885

[6] Lamprecht B McBurnie MA Vollmer WM Gudmundsson G Welte T Nizankowska-Mogilnicka E. COPD in never smokers: results from the population-based burden of obstructive lung disease study. Chest. (2011) 139:752–63. 10.1378/chest.10-1253 20884729 PMC3168866

[7] Salvi SS Barnes PJ. Chronic obstructive pulmonary disease in non-smokers. Lancet. (2009) 374:733–43. 10.1016/S0140-6736(09)61303-9 19716966

[8] Smith M Li L Augustyn M Kurmi O Chen J Collins R. Prevalence and correlates of airflow obstruction in ~317,000 never-smokers in china. Eur Respir J. (2014) 44:66–77. 10.1183/09031936.00152413 24603814 PMC4076527

[9] World Health Organization. Fuel for Life: Household Energy and Health (2006). Available online at: https://www.who.int/airpollution/publications/fuelforlife.pdf?ua=1 (accessed November 30, 2021).

[10] Balmes JR. Household air pollution from domestic combustion of solid fuels and health. J Allergy Clin Immunol. (2019) 143:1979–87. 10.1016/j.jaci.2019.04.016 31176380

[11] Gordon SB Bruce NG Grigg J Hibberd PL Kurmi OP Lam KB. Respiratory risks from household air pollution in low and middle income countries. Lancet Respir Med. (2014) 2:823–60. 10.1016/S2213-2600(14)70168-7 25193349 PMC5068561

[12] Sood A Assad NA Barnes PJ Churg A Gordon SB Harrod KS. ERS/ATS workshop report on respiratory health effects of household air pollution. Eur Respir J. (2018) 51:1700698. 10.1183/13993003.00698-2017 29301918 PMC7418845

[13] Ho KF Chang CC Tian L Chan CS Musa Bandowe BA Lui KH. Effects of polycyclic aromatic compounds in fine particulate matter generated from household coal combustion on response to EGFR mutations *in vitro*. Environ Pollut. (2016) 218:1262–69. 10.1016/j.envpol.2016.08.084 27613327

[14] Simkovich SM Goodman D Roa C Crocker ME Gianella GE Kirenga BJ. The health and social implications of household air pollution and respiratory diseases. NPJ Prim Care Respir Med. (2019) 29:12. 10.1038/s41533-019-0126-x 31028270 PMC6486605

[15] Wang C Xu J Yang L Xu Y Zhang X Bai C. Prevalence and risk factors of chronic obstructive pulmonary disease in China (the China pulmonary health [CPH] study): a national cross-sectional study. Lancet. (2018) 391:1706–17. 10.1016/S0140-6736(18)30841-9 29650248

[16] Duan X Jiang Y Wang B Zhao X Shen G Cao S. Household fuel use for cooking and heating in China: results from the first Chinese environmental exposure-related human activity patterns survey (CEERHAPS). Appl Energy. (2014) 136:692–703. 10.1016/j.apenergy.2014.09.066

[17] Guo Y Xing Z Shan G Janssens JP Sun T Chai D. Prevalence and risk factors for COPD at high altitude: a Large cross-sectional survey of subjects living between 2,100-4,700 m above sea level. Front Med. (2020) 7:581763. 10.3389/fmed.2020.581763 33344472 PMC7744817

[18] Miller MR Hankinson J Brusasco V Burgos F Casaburi R Coates A. Standardisation of spirometry. Eur Respir J. (2005) 26:319–38. 10.1183/09031936.05.00034805 16055882

[19] Quanjer PH Stanojevic S Cole TJ Baur X Hall GL Culver BH. Multi-ethnic reference values for spirometry for the 3-95-yr age range: the global lung function 2012 equations. Eur Respir J. (2012) 40:1324–43. 10.1183/09031936.00080312 22743675 PMC3786581

[20] Xiao D Chen Z Wu S Huang K Xu J Yang L. Prevalence and risk factors of small airway dysfunction, and association with smoking, in China: findings from a national cross-sectional study. Lancet Respir Med. (2020) 8:1081–93. 10.1016/S2213-2600(20)30155-7 32598906

[21] Survey NHI. Adult Tobacco Use Information (2017). Available online at: https://www.cdc.gov/nchs/nhis/tobacco/tobacco_glossary.htm (accessed November 30, 2021).

[22] Adeloye D Chua S Lee C Basquill C Papana A Theodoratou E. Global and regional estimates of COPD prevalence: systematic review and meta-analysis. J Glob Health. (2015) 5:020415. 10.7189/jogh.05.020415 26755942 PMC4693508

[23] Lamprecht B Schirnhofer L Kaiser B Buist S Studnicka M. Non-reversible airway obstruction in never smokers: results from the Austrian BOLD study. Respir Med. (2008) 102:1833–8. 10.1016/j.rmed.2008.07.007 18722100

[24] Hagstad S Ekerljung L Lindberg A Backman H Rönmark E Lundbäck B. COPD among non-smokers - report from the obstructive lung disease in Northern Sweden (OLIN) studies. Respir Med. (2012) 106:980–8. 10.1016/j.rmed.2012.03.010 22498109

[25] Tan WC Sin DD Bourbeau J Hernandez P Chapman KR Cowie R. Characteristics of COPD in never-smokers and ever-smokers in the general population: results from the canCOLD study. Thorax. (2015) 70:822–9. 10.1136/thoraxjnl-2015-206938 26048404

[26] Siddharthan T Grigsby MR Goodman D Chowdhury M Rubinstein A Irazola V. Association between household air pollution exposure and chronic obstructive pulmonary disease outcomes in 13 low- and middle-Income country settings. Am J Respir Crit Care Med. (2018) 197:611–20. 10.1164/rccm.201709-1861OC 29323928 PMC6005243

[27] Chan KH Kurmi OP Bennett DA Yang L Chen Y Tan Y. Solid fuel use and risks of respiratory diseases. A cohort study of 280,000 Chinese never-smokers. Am J Respir Crit Care Med. (2019) 199:352–61. 10.1164/rccm.201803-0432OC 30235936 PMC6363974

[28] Raju S Keet CA Paulin LM Matsui EC Peng RD Hansel NN. Rural residence and poverty are independent risk factors for chronic obstructive pulmonary disease in the United States. Am J Respir Crit Care Med. (2019) 199:961–9. 10.1164/rccm.201807-1374OC 30384774 PMC6467317

[29] McDonough JE Yuan R Suzuki M Seyednejad N Elliott WM Sanchez PG. Small-airway obstruction and emphysema in chronic obstructive pulmonary disease. N Engl J Med. (2011) 365:1567–75. 10.1056/NEJMoa1106955 22029978 PMC3238466

[30] Hogg JC Chu F Utokaparch S Woods R Elliott WM Buzatu L. The nature of small-airway obstruction in chronic obstructive pulmonary disease. N Engl J Med. (2004) 350:2645–53. 10.1056/NEJMoa032158 15215480

[31] Zhao D Zhou Y Jiang C Zhao Z He F Ran P. Small airway disease: a different phenotype of early stage COPD associated with biomass smoke exposure. Respirology. (2018) 23:198–205. 10.1111/resp.13176 28906034

[32] Fernandes L Gulati N Fernandes Y Mesquita AM Sardessai M Lammers JJ. Small airway imaging phenotypes in biomass- and tobacco smoke-exposed patients with COPD. ERJ Open Res. (2017) 3:00124–2016. 10.1183/23120541.00124-2016 28828380 PMC5555765

[33] Regalado J Pérez-Padilla R Sansores R Páramo Ramirez JI Brauer M Paré P. The effect of biomass burning on respiratory symptoms and lung function in rural Mexican women. Am J Respir Crit Care Med. (2006) 174:901–5. 10.1164/rccm.200503-479OC 16799080

[34] Pope D Diaz E Smith-Sivertsen T Lie RT Bakke P Balmes JR. Exposure to household air pollution from wood combustion and association with respiratory symptoms and lung function in nonsmoking women: results from the RESPIRE trial, Guatemala. Environ Health Perspect. (2015) 123:285–92. 10.1289/ehp.1408200 25398189 PMC4384202

[35] Capistrano SJ van Reyk D Chen H Oliver BG. Evidence of biomass smoke exposure as a causative factor for the development of COPD. Toxics. (2017) 5:36. 10.3390/toxics5040036 29194400 PMC5750564

[36] Zhou Y Zou Y Li X Chen S Zhao Z He F. Lung function and incidence of chronic obstructive pulmonary disease after improved cooking fuels and kitchen ventilation: a 9-year prospective cohort study. PLoS Med. (2014) 11:e1001621. 10.1371/journal.pmed.1001621 24667834 PMC3965383

[37] Chapman RS He X Blair AE Lan Q. Improvement in household stoves and risk of chronic obstructive pulmonary disease in Xuanwei, China: retrospective cohort study. BMJ. (2005) 331:1050. 10.1136/bmj.38628.676088.55 16234255 PMC1283181

[38] World Health Organization. Household Air Pollution (2016). Available online at: https://www.who.int/data/gho/data/themes/topics/topic-details/GHO/household-air-pollution (accessed November 30, 2021).

[39] Perez-Padilla R Fernandez R Lopez Varela MV Montes de Oca M Muiño A Tálamo C. Airflow obstruction in never smokers in five Latin American cities: the PLATINO study. Arch Med Res. (2012) 43:159–65. 10.1016/j.arcmed.2012.03.007 22475778

[40] Kojima S Sakakibara H Motani S Hirose K Mizuno F Ito M. Effects of smoking and age on chronic obstructive pulmonary disease in Japan. J Epidemiol. (2005) 15:113–7. 10.2188/jea.15.113 16141629 PMC7851068

