# Supplementary material for: Solid Fuel Exposure and Chronic Obstructive Pulmonary Disease in Never-Smokers
Source: Front Med (Lausanne). 2021 Dec 22;8:757333. doi: 10.3389/fmed.2021.757333 (PMC8727436; doi:10.3389/fmed.2021.757333)
Supplement: Supplementary file 1 [file Data_Sheet_1.PDF]

Supplementary Table 1. Severity of COPD according to GOLD grades.

	Overall (n = 667)		Men (n = 236)		Women (n = 431)	
	Cases (n)	Proportion	Cases (n)	Proportion	Cases (n)	Proportion
GOLD stage I(mild)	273	40.9%	94	39.8%	179	41.5%
GOLD stage II (moderate)	326	48.9%	105	44.5%	221	51.3%
GOLD stage III-IV (severe-very severe)	68	10.2%	37	15.7%	31	7.2%

COPD, chronic obstructive pulmonary disease; GOLD, Global Initiative for Chronic Obstructive Pulmonary Disease.

Supplementary Table 2. Distributions of typical symptoms in the normal population and COPD patients by GOLD grades.

Symptoms	Status	Normal n=5604	GOLD I n=273	GOLD II n=326	GOLD III-IV n=68	Overall COPD n=667
Chronic cough	with	451(8.05)	34(12.45)	38(11.66)	10(14.71)	82(12.29)
	without	5079(90.63)	237(86.81)	287(88.04)	58(85.29)	582(87.26)
Chronic phlegm	with	373(6.66)	31(11.36)	31(9.51)	12(17.65)	74(11.09)
	without	5155(91.99)	240(87.91)	294(90.18)	56(82.35)	590(88.46)
Recurrent wheezing	with	211(3.77)	15(5.49)	22(6.75)	8(11.76)	45(6.75)
	without	5316(94.86)	256(93.77)	303(92.94)	60(88.24)	619(92.80)
Dyspnea in daily life	with	1078(19.24)	89(32.60)	68(20.86)	20(29.41)	177(26.54)
	without	4453(79.46)	182(66.67)	275(84.36)	48(70.59)	487(73.01)
At least one of the above symptoms	with	1456(25.98)	112(41.03)	93(28.53)	25(36.76)	230(34.48)
	without	4148(74.02)	161(58.97)	233(71.47)	43(63.24)	437(65.52)

COPD, chronic obstructive pulmonary disease; GOLD, Global Initiative for Chronic Obstructive Pulmonary Disease.

Supplementary Figure 1. Flow of participants through the study.

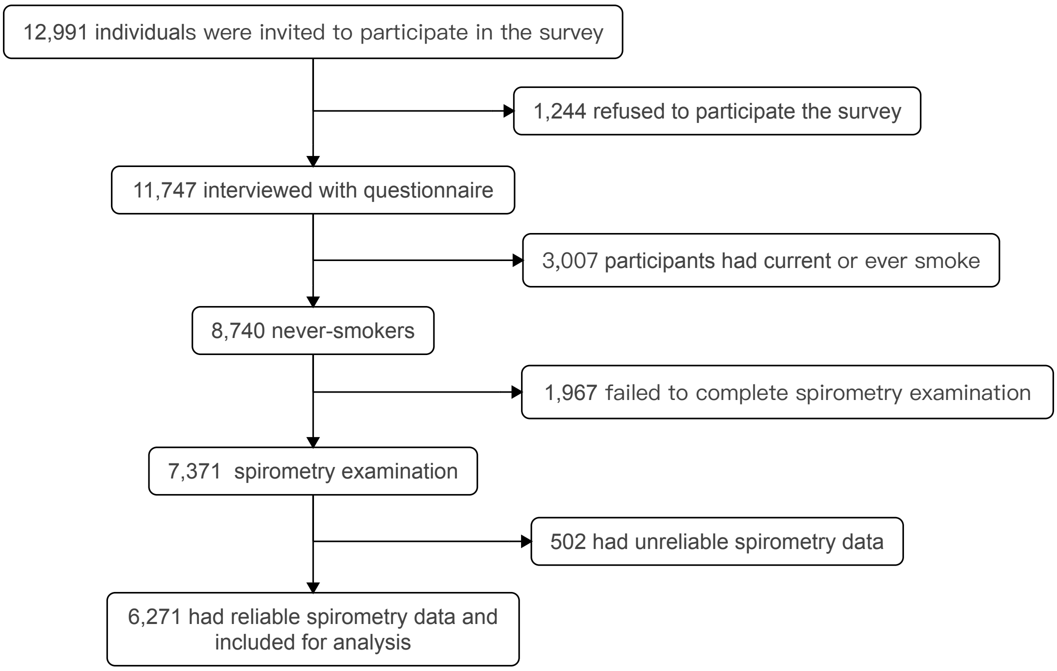

Supplementary Figure 2. Prevalence and corresponding 95%CI of COPD by age-stratified.
a) overall; b) men; c) women

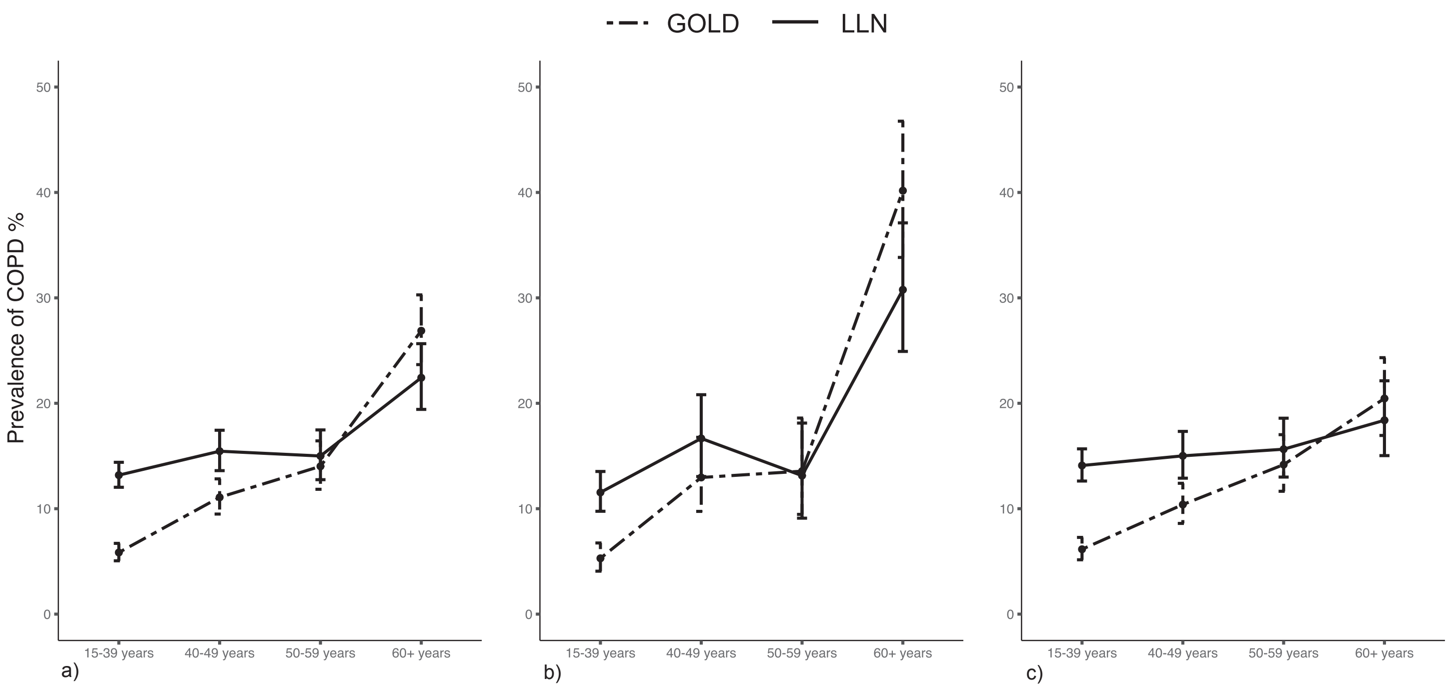

COPD, chronic obstructive pulmonary disease; CI, confidence interval. Bars represent the prevalence and error bars the 95%CI.
